# Obesity, Twin Pregnancy, and the Role of Assisted Reproductive Technology

**DOI:** 10.1001/jamanetworkopen.2023.50934

**Published:** 2024-01-09

**Authors:** Jeffrey N. Bone, K. S. Joseph, Laura A. Magee, Li Qing Wang, Sid John, Mohamed A. Bedaiwy, Chantal Mayer, Sarka Lisonkova

**Affiliations:** 1 Department of Obstetrics and Gynaecology, University of British Columbia, Children’s and Women’s Hospital and Health Centre, Vancouver, British Columbia, Canada; 2 Biostatistics, British Columbia Children’s Hospital Research Institute, Vancouver, British Columbia, Canada; 3 School of Population and Public Health, University of British Columbia, Vancouver, British Columbia, Canada; 4 Department of Women and Children’s Health, School of Life Course Sciences, Faculty of Life Sciences and Medicine, King’s College London, London, United Kingdom

## Abstract

**Importance:**

The prevalence of overweight and obesity (body mass index [BMI] ≥25) has increased globally, and high BMI has been linked to higher rates of twin birth. However, evidence from large population-based studies is lacking; the issue needs careful study, as women with obesity are also more likely to use assisted reproductive technology (ART), which frequently results in twin pregnancy.

**Objective:**

To examine the association between BMI and twin birth and the role of ART as a potential mediator in this association.

**Design, Setting, and Participants:**

This retrospective cohort study included all live births and stillbirths with gestational age of 20 weeks or longer in British Columbia, Canada, from 2008 to 2020, using data from the British Columbia Perinatal Database Registry. Data analysis was conducted from November 2022 to June 2023.

**Exposures:**

Prepregnancy BMI, calculated as weight in kilograms divided by height in meters squared, and use of ART.

**Main Outcomes and Measures:**

The study assessed whether prepregnancy BMI is associated with the rate of twin vs singleton delivery and whether this association is explained by the differential use of ART in women with obesity.

**Results:**

A total of 524 845 deliveries at 20 weeks’ or longer gestation occurred in British Columbia during the study period, and 392 046 women had complete data on prepregnancy BMI. The median (IQR) age was 31.4 (27.7-35.0) years, approximately half were nulliparous (243 443 [46.4%]) and less than 10% smoked during pregnancy (36 894 [7.1%]). Overall, 8295 women had a twin delivery (15.8 per 1000 deliveries), and rates per 1000 deliveries by prepregnancy BMI categories were 11.9 (underweight), 15.1 (normal), 16.0 (overweight), 16.0 (obesity class I), 16.7 (obesity class II), and 18.9 (obesity class III). After adjustment for other covariates, women with underweight had relatively 16% fewer twins compared with women with normal BMI (adjusted risk ratio [aRR], 0.84; 95% CI, 0.74-0.95), while women with overweight, class I obesity, class II obesity, and class III obesity had 14% (aRR, 1.14; 95% CI, 1.07-1.21), 16% (aRR, 1.16; 95% CI, 1.06-1.27), 17% (aRR, 1.17; 95% CI, 1.02-1.34), and 41% higher rates (aRR, 1.41; 95% CI, 1.19-1.66), respectively. The proportion of women who conceived by ART increased with increasing BMI, and ART was associated with nearly a 12-fold higher rate of twin delivery (aRR, 11.80; 95% CI 11.10-12.54). ART explained about a quarter of the association between obesity class I and II and twin delivery (eg, obesity class I, 23% mediated; 95% CI, 7%-39% mediated), but none of this association was mediated by ART in women with class III obesity.

**Conclusions and relevance:**

In this cohort study of 524 845 births, the rate of twin birth increased with increasing prepregnancy BMI. In women with a BMI between 30 and 40, approximately one-quarter of this association was explained by higher use of ART; however, there was no evidence of such mediation in women with BMI of 40 or greater.

## Key Points

**Question:**

Does increasing prepregnancy body mass index (BMI) increase the rate of twin pregnancy, and is this associated with higher rates of assisted reproductive technology (ART)?

**Findings:**

In this cohort study of 524 845 live and still births, the rate of twin pregnancy increased consistently with increasing BMI, with the highest rates in those with a BMI of 40 or greater. The association between increased twinning in those with BMIs between 25 and 30 was partially mediated by greater use of ART.

**Meaning:**

In this study, the rate of twin deliveries increased with increasing BMI and part of this association was explained by higher use of ART in women with class I and II obesity.

## Teaser

This cohort study examines the association between body mass index (BMI) and twin birth as well as the role of assisted reproductive technologies (ART) as a potential mediator in British Columbia, Canada.

## Introduction

The global rate of twin deliveries has increased from 9.1 to 12.0 per 1000 deliveries between 1980 to 1985 and 2010 to 2015.^1^ Most of this increase was due to increases in dizygotic twinning, as the rate of monozygotic twins has been broadly consistent worldwide and stable over time.^2,3^ Temporal increases in twin deliveries represent a clinical and public health concern, as twin pregnancy is associated with elevated risks of maternal as well as fetal and infant complications.^4,5,6^

A primary reason for the secular increase in twinning rates has been an increased use of assisted reproductive technology (ART).^2,7,8,9,10^ In the United States, ART contributed to an estimated 10.6% of multiples (eg, twins and triplets), and 16.8% of infants conceived with ART in 2019 were multiples.^11,12^ Twins conceived by ART are mostly dizygotic, although there is some evidence that monozygotic twinning is also increased by ART.^13^ Another contributor to the increased rate of dizygotic twinning is a temporal increase in maternal age, as advanced maternal age is associated with a 25% to 30% increased risk of twin pregnancies.^8,14,15^ Additional risk factors for twinning include higher parity,^14,16,17^ tall maternal stature,^17,18^ smoking,^19^ and race other than White.^20,21^

The proportion of women with overweight and obesity has increased in most countries, affecting 30% to 50% of women of reproductive age.^22,23,24,25^ This change is concerning as obesity is one of the most common and potentially modifiable risk factors for adverse pregnancy outcomes.^26,27,28,29^ Previous studies from the United States (1959-1966) and Denmark (1998-2001) have shown an association between obesity and twinning rates, particularly with regard to dizygotic twins.^18,30^ Although the mechanism underlying this association is unclear, it is possible that the higher rates of twins among women with obesity are linked to higher rates of ART, as women with overweight and obesity are more likely to have subfertility compared with those with normal body mass index (BMI; calculated as weight in kilograms divided by height in meters squared).^31,32^

The objective of this study was to examine the association between high prepregnancy BMI and twin delivery in a contemporary cohort of Canadian women. Additionally, we assessed the extent to which this association is mediated by ART use.

## Methods

### Data Source and Study Population

This was a retrospective cohort study of all women who delivered a singleton or twin live or stillbirth at 20 weeks’ or longer gestation in British Columbia, Canada, between April 2008 and March 2020. Ethics approval was obtained from the University of British Columbia–Children’s and Women’s Hospital and Health Centre of British Columbia Research Ethics Board. Data are routinely collected as part of routine provincial data collection and are not subject to individual consent. This study was conducted in accordance with the Strengthening the Reporting of Observational Studies in Epidemiology (STROBE) reporting guideline.

Women who delivered at less than 20 weeks’ gestation, those with missing information on gestational age and plurality, and those with pregnancy termination were excluded. Data were obtained from the British Columbia Perinatal Database Registry (BCPDR), which has contained individual level data on more than 99% of all deliveries in British Columbia since 2000, including home deliveries.^33^ All analyses were based on women, and the delivery (singleton or twin) constituted the unit of analysis (irrespective of whether the delivery resulted in 1 live birth, 2 live births, 2 stillbirths or 1 live birth and 1 stillbirth). Validation studies of BCPDR data, based on reabstraction of medical records, have showed high accuracy of the collected data.^34^ The BCPDR includes information about maternal demographic characteristics, prepregnancy BMI, chronic medical conditions, and pregnancy morbidity as well as details about delivery and newborn hospitalizations.

### Exposure, Mediator, and Outcome

The primary exposure was prepregnancy BMI, based on self-reported height and prepregnancy weight and categorized as follows: underweight (<18.5), normal BMI (18.5 to 24.9), overweight (25.0 to 29.9), and obesity class I (30.0 to 34.9), class II (35.0 to 39.9), and class III (≥40.0). The primary outcome was twin delivery. To further explore the possible association between prepregnancy BMI and twin delivery, we conducted a mediation analysis with ART conception as the mediator. ART was defined as the use of in vitro fertilization (IVF) to conceive; this information was based on maternal recall and was abstracted from medical records to BCPDR. Mediation analyses allow for the disentangling of possible pathways by which an exposure is associated with an outcome, thereby enabling an answer to the question, “What would the rate of twins be in women with obesity if they used ART at the same rate as women of normal BMI?” To estimate unbiased associations, mediation analyses require adjustment for all confounders of all pairs of the exposure, mediator, and outcome relationships. Confounders were identified from previous studies on twinning and included maternal age, parity, smoking during pregnancy (self-reported), and maternal height. eFigure 1 in Supplement 1 illustrates the assumed connections between these variables. We found evidence of a reduction in the rate of twin deliveries among ART pregnancies during our study period (eFigure 2 in Supplement 1). Therefore, we included fiscal year as a confounder.

### Statistical Analysis

Participant characteristics were summarized across prepregnancy BMI categories. Robust Poisson regression was used to estimate unadjusted and adjusted (for previously described confounders) rate ratios for the use of ART by BMI group. Rates of twin delivery were calculated per 1000 deliveries, and the rates were compared between BMI categories, collectively and stratified by ART conception. We further summarized twin delivery rate by continuous BMI descriptively using a univariate logistic regression model with a natural spline with 5 knots at the quintiles of prepregnancy BMI distribution.

Mediation analyses used the regression-based methods of Valeri and VanderWeele^35^ to estimate the total association of prepregnancy BMI with twin delivery and to decompose this into the indirect effect through ART conception and the remaining direct effect not mediated through ART.^36^ Log-binomial regression models were used to estimate total effects, natural direct, and indirect effects (on the rate ratio scale) and the proportion mediated by ART. Standard errors were computed using the Delta method, and models included an interaction between BMI and ART.^36,37^

We used the rate ratios for the total associations to calculate population attributable fractions (PAFs) to estimate proportion of twin births attributable to prepregnancy overweight and obesity. Confidence intervals for PAFs were calculated using simulation.^38^ We computed E-values for the sensitivity of our results to unmeasured confounding (eg, by genetic factors or race and ethnicity). E-values represent the minimum strength of the association (on the rate ratio scale) that an unmeasured confounder would have to have with both prepregnancy BMI and twin delivery to render the observed association null.^39^

Multiple imputation was used for missing values for BMI in all analyses. Results were pooled from the 20 multiply imputed data sets using Rubin’s rules.^40^

We conducted several sensitivity analyses, including restriction to opposite sex twins (as a proxy for chorionicity), complete case analysis, deterministic imputation, adjustment for BMI misclassification, and possible left-truncation bias. Further details and rationale of all sensitivity analyses are provided in the eAppendix in Supplement 1. Analyses were conducted using R statistical software version 4.3.0 (Project for Statistical Computing), with mediation analysis performed using CMAverse.^41^

## Results

A total of 524 845 deliveries at 20 weeks’ or longer gestation occurred in British Columbia from 2008 to 2020. The median (IQR) age was 31.4 (27.7-35.0) years, approximately half were nulliparous (243 443 [46.4%]) and less than 10% smoked during pregnancy (36 894 [7.1%]). A total of 392 046 women (74.7%) had complete data on prepregnancy BMI; of these, 22 396 women (5.7%) had underweight, 231 583 (59.1%) had normal BMI, 83 887 (21.4%) had overweight, and 33 363 (8.5%), 13 308 (3.4%), and 7609 (1.9%) had obesity class I, II, and III, respectively (eFigure 3 in Supplement 1).

Maternal age and height were similar between all BMI groups, except for women with underweight, who were relatively younger. Women with obesity were more likely to have higher parity, while those with underweight and obesity were more likely than those with normal BMI to smoke during pregnancy (Table 1). Women with missing BMI were less likely to be nulliparous and had a slightly higher rate of twin delivery (eTable 1 in Supplement 1).

**Table 1.** Demographic Characteristics of Women by Prepregnancy Body Mass Index Category, British Columbia, Canada, 2008-2020

Characteristic	Women, No. (%)					
	Underweight (n = 22 396)	Normal (n = 231 583)	Overweight (n = 83 887)	Obesity class		
				I (n = 33363)	II (n = 13 308)	III (n = 7609)
Maternal age, y						
Median (IQR)	30.1 (26.3-33.7)	31.6 (28.1-35.0)	31.6 (28.0-35.1)	31.3 (27.6-34.9)	31.1 (27.4-34.7)	31.4 (27.8-34.9)
<20	779 (3.5)	4443 (1.9)	1276 (1.5)	455 (1.4)	166 (1.3)	76 (1.0)
20-24	3239 (14.5)	21 908 (9.5)	8467 (10.1)	3855 (11.6)	1592 (12.0)	806 (10.6)
25-34	14 394 (64.3)	146 819 (63.4)	52 146 (62.2)	20 744 (62.4)	8381 (63.0)	4827 (63.4)
35-39	3314 (14.8)	48 101 (20.8)	17 769 (21.2)	6726 (20.2)	2590 (19.5)	1553 (20.4)
≥40	670 (2.99)	10 312 (4.5)	4229 (5.0)	1483 (4.5)	579 (4.4)	347 (4.6)
Parity						
Nulliparous	12 554 (56.1)	117 770 (50.9)	37 211 (44.4)	13 667 (41.1)	5355 (40.2)	3000 (39.4)
1	7199 (32.1)	81 015 (35.0)	30 935 (36.9)	12 218 (36.7)	4902 (36.8)	2738 (36.0)
≥2	2642 (11.8)	32 780 (14.2)	15 728 (18.8)	7375 (22.2)	3050 (22.9)	1869 (24.6)
Maternal height, cm						
Median (IQR)	165 (160-170)	165 (160-170)	164 (160-169)	164 (160-169)	165 (160-170)	165 (160-170)
<165	10 631 (47.5)	114 967 (49.6)	42 239 (50.4)	16 695 (50.2)	6299 (47.3)	3705 (48.7)
165-168	5852 (26.1)	55 105 (23.8)	19 807 (23.6)	7900 (23.8)	3366 (25.3)	1889 (24.8)
169-172	2507 (11.2)	27 675 (12.0)	10 043 (12.0)	3893 (11.7)	1669 (12.5)	928 (12.2)
≥173	3406 (15.2)	33 836 (14.6)	11 798 (14.1)	4775 (14.4)	1974 (14.8)	1087 (14.3)
Smoking						
Yes	1727 (7.7)	12 954 (5.6)	6159 (7.3)	3241 (9.7)	1462 (11.0)	881 (11.6)
No	20 669 (92.3)	218 629 (94.4)	77 728 (92.7)	30 022 (90.3)	11 846 (89.0)	6728 (88.4)

The frequency of ART conception (per 1000 deliveries) was lowest among women with underweight (22.1) and women with class III obesity (20.4) and was similar in other groups (rates between 30 and 32); however, after adjustment for confounders, compared with women with normal BMI, women who had overweight, obesity class I, and obesity class II had 9%, 23% and 26% higher rates of ART use, respectively (overweight: adjusted rate ratio [aRR], 1.09; 95% CI, 1.05-1.14; obesity class I: aRR, 1.23; 95% CI, 1.16-1.31; obesity class II: aRR, 1.26; 95% CI, 1.15-1.38). These results were similar in complete case analysis and after multiple imputation analyses (Table 2).

**Table 2.** Rates, RRs, and aRRs for Use of ART by Prepregnancy BMI Category

Prepregnancy BMI	Total births, No.	ART births, No. (per 1000)	Complete case analysis		Multiple imputation^a^	
			RR (95% CI)	aRR (95% CI)^b^	RR (95% CI)	aRR (95% CI)^b^
Underweight	22 396	495 (22.1)	0.75 (0.69-0.82)	0.92 (0.84-1.00)	0.80 (0.73-0.88)	0.93 (0.85-1.02)
Normal	231 583	6823 (29.5)	1 [Reference]	1 [Reference]	1 [Reference]	1 [Reference]
Overweight	83 887	2570 (30.6)	1.04 (0.99-1.09)	1.09 (1.04-1.14)	1.04 (0.99-1.08)	1.09 (1.05-1.14)
Obesity class I	33 263	1054 (31.7)	1.08 (1.01-1.15)	1.25 (1.18-1.33)	1.07 (1.00-1.14)	1.23 (1.16-1.31)
Obesity class II	13 308	426 (32.0)	1.09 (0.99-1.20)	1.30 (1.18-1.43)	1.07 (0.97-1.18)	1.26 (1.15-1.38)
Obesity class III	7609	155 (20.4)	0.69 (0.59-0.81)	0.82 (0.71-0.96)	0.77 (0.65-0.90)	0.91 (0.78-1.06)

Abbreviations: aRR, adjusted rate ratio; ART, assisted reproductive technology; BMI, body mass index (calculated as weight in kilograms divided by height in meters squared); RR, rate ratio.

^a^ Results pooled from 20 imputed data sets.

^b^ Adjusted for maternal height, age, smoking status, parity, and fiscal year.

The overall rate of twin delivery was 15.8 per 1000 deliveries. Compared with women with normal BMI (14.4 per 1000 deliveries), women with overweight and obesity class I and II had moderately higher rates of twin delivery (16.0, 16.0, and 16.7 per 1000 deliveries, respectively), and women with underweight had lower rates (11.3 per 1000 deliveries) (Table 3 and Figure). Women with class III obesity had the highest rates of twin delivery (18.9 per 1000 deliveries). Table 3 and eFigure 4 in Supplement 1 show these rates stratified by use of ART with similar results to those previously described, with the exception women with of class III obesity who conceived by ART, among whom the rates of twin birth were similar to other groups. Overall, ART use was associated with a nearly 12-fold increased rate of twinning (aRR, 11.80; 95% CI 11.10-12.54).

**Table 3.** Twin Delivery Rates by Prepregnancy BMI Category and Use of ART

Prepregnancy BMI	Overall		ART		Non-ART	
	Total births, No.	Twin births, No. (per 1000)	Total births, No.	Twin births, No. (per 1000)	Total births, No.	Twin births, No. (per 1000)
Underweight	22 396	253 (11.3)	495	65 (131.3)	21 901	188 (8.58)
Normal	231 583	3323 (14.4)	6823	977 (143.2)	224 760	2346 (10.44)
Overweight	83 887	1340 (16.0)	2570	363 (141.3)	81 317	977 (12.01)
Obese I	33 263	531 (16.0)	1054	134 (127.1)	32 209	397 (12.33)
Obese II	13 308	222 (16.7)	426	63 (147.9)	12 882	159 (12.34)
Obese III	7609	144 (18.9)	155	23 (148.4)	7454	121 (16.23)

Abbreviations: ART, assisted reproductive technology; BMI, body mass index (calculated as weight in kilograms divided by height in meters squared).

**Figure.**
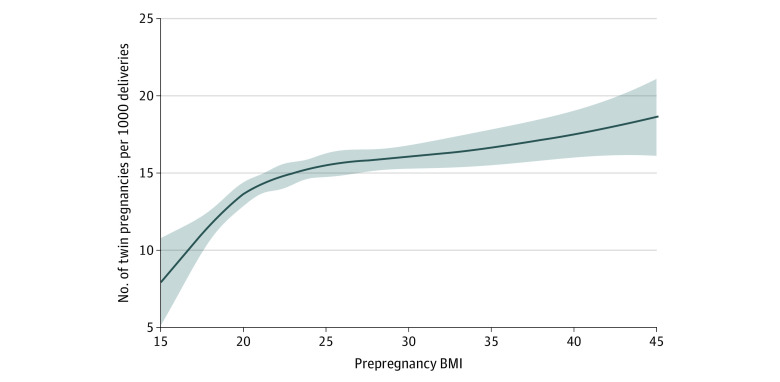
Association Between Prepregnancy Body Mass Index (BMI) and Rate of Twin Delivery BMI is calculated as weight in kilograms divided by height in meters squared.

The adjusted rate of twin delivery increased with increasing BMI; women with overweight and obesity class I, II, and III had 16%, 17%, and 41%, higher rates of twin delivery than women with normal BMI (overweight: aRR, 1.14; 95% CI, 1.07-1.21; obesity class I: aRR, 1.16; 95% CI, 1.06-1.27; obesity class II: aRR, 1.17; 95% CI, 1.02-1.34; obesity class III: aRR, 1.41; 95% CI, 1.19-1.66), while women with underweight had a lower rate (aRR, 0.84; 95% CI 0.74-0.95) (Table 3). Mediation analysis showed that an increasing proportion of the association between higher BMI and twinning was mediated by increased use of ART: 11% (95% CI, 3%-18%) mediated in women with overweight; 23% (95% CI, 7%-39%) in women with class I obesity; and 27% (95% CI, 0%-54%) in women with class II obesity. However, the trend was reversed in women with class III obesity, with no evidence of mediation by ART (Table 4). Overall, 4% of twin births were attributable to above-normal prepregnancy BMI (overweight and obesity: PAF, 4.0%; 95% CI, 1.9%-6.0%). The association between obesity and twin birth was relatively sensitive to unmeasured confounding with all E-values less than 2.20 (Table 4).

**Table 4.** Adjusted Associations Between Prepregnancy BMI Category and Twin Delivery and Mediation Analysis by ART Conception

Prepregnancy BMI	aRR (95 CI)^a^			Proportion mediated, % (95% CI)	E-value for total effect^b^
	Total effect	Natural direct effect	Natural indirect effect		
**All twins**					
Underweight	0.84 (0.74 to 0.95)	0.85 (0.75 to 0.96)	0.99 (0.98 to 1.00)	6 (−2 to 13)	1.67
Normal	1 [Reference]	1 [Reference]	1 [Reference]	NA	NA
Overweight	1.14 (1.07 to 1.21)	1.12 (1.05 to 1.19)	1.01 (1.01 to 1.02)	11 (3 to 18)	1.54
Obesity class I	1.16 (1.06 to 1.27)	1.12 (1.03 to 1.23)	1.03 (1.02 to 1.04)	23 (7 to 39)	1.59
Obesity class II	1.17 (1.02 to 1.34)	1.13 (0.98 to 1.29)	1.04 (1.02 to 1.06)	27 (0 to 54)	1.61
Obesity class III	1.41 (1.19 to 1.66)	1.44 (1.22 to 1.69)	0.98 (0.96 to 1.00)	−7 (−15 to 0)	2.17
**Opposite sex twins only**					
Underweight	0.73 (0.58 to 0.91)	0.74 (0.69 to 0.93)	0.98 (0.96 to 1.01)	5 (−3 to 13)	2.08
Normal	1 [Reference]	1 [Reference]	1 [Reference]	NA	NA
Overweight	1.22 (1.09 to 1.35)	1.19 (1.07 to 1.33)	1.02 (1.01 to 1.03)	11 (3 to 19)	1.73
Obesity class I	1.33 (1.15 to 1.54)	1.27 (1.10 to 1.48)	1.05 (1.03 to 1.07)	18 (7 to 30)	1.99
Obesity class II	1.47 (1.19 to 1.81)	1.39 (1.13 to 1.72)	1.05 (1.02 to 1.08)	16 (5 to 27)	2.30
Obesity class III	1.64 (1.26 to 2.13)	1.68 (1.29 to 2.18)	0.97 (0.95 to 1.00)	−7 (−15 to 2)	2.67

Abbreviations: aRR, adjusted risk ratio; ART, assisted reproductive technology; BMI, body mass index (calculated as weight in kilograms divided by height in meters squared); NA, not applicable.

^a^ Effects are estimated from mediation analyses pooled across 20 multiply imputed data sets. aRRs adjusted for maternal height, age, smoking status, parity, and fiscal year.

^b^ E-values represent strength of unmeasured confounder (on rate ratio scale) needed to bring the point estimate for total effects to 1.0.

### Sensitivity Analyses

Approximately two-thirds of twin deliveries included same sex twins (eTable 2 in Supplement 1). Among pregnancies with opposite sex twins, the rate of ART use was 37% compared with 23% in pregnancies with same sex twins. Sensitivity analyses restricted to opposite sex twin births showed that the overall association between obesity and twin birth was stronger than in all twin births. The results of mediation analysis were slightly attenuated compared with the primary results (Table 4).

Complete-case analyses yielded nearly identical results to analyses using multiple imputation (eTable 3 in Supplement 1). In scenarios examining worst-case biases for the proportion mediated, we found large variation in the role of ART, varying from near null to greater than 50% mediation for the association between BMI and twinning. In these scenarios, and the 2 corresponding to worst case biases for the total association, the dose-response association between higher BMI and higher rates of twinning was maintained (eTable 4 in Supplement 1). The association between higher BMI and twinning was robust to possible measurement bias corresponding to underreporting of BMI; however, such correction for measurement error attenuated the proportion mediated by ART (eTable 5 in Supplement 1). Compared with the primary results, the quantitative bias analysis, assuming moderately different rates of pregnancy loss before 20 weeks’ gestation between women with normal BMI and obesity, yielded slightly stronger associations between high BMI and twinning (eg, for class III obesity, bias-adjusted RR, 1.46; 95% CI, 1.21-1.77) (eTable 6 in Supplement 1).

## Discussion

In this population-based cohort study, we found a relatively small excess of twin deliveries in women who had overweight and obesity prior to pregnancy compared with women with normal BMI. Only 4% of twin deliveries among women with overweight and obesity were attributable to elevated BMI. Women with high BMI had a higher adjusted frequency of ART conception. ART accounted for about a quarter of the association between BMI and twinning in women with BMI between 30.0 and 39.9; however, in women with BMI of 40 or greater, who had the highest rates of twin birth, ART did not play any mediating role. We found a stronger association between BMI and opposite-sex twin delivery, but the proportion mediated by ART conception was smaller.

Understanding the potential causes of twin pregnancy and delivery is essential from both clinical and public health perspectives, as women with twin pregnancy are more likely to experience complications, including severe maternal morbidity, preterm birth, and stillbirth.^4,5,6^ Two large studies have shown elevated twinning rates in women with increased BMI.^18,30^ A cohort study from Denmark found that women with obesity had 44% higher odds of twin birth and 62% higher odds of opposite-sex twins.^18^ The second study examined twin pregnancies in the United States in 1959 to 1966, before the widespread use of ART, and showed a 2-fold increase in the twin rate in dizygotic twins among women with higher BMI, but no association between BMI and the rate of monozygotic twins.^30^ While our study also found associations between obesity and twin birth, they were weaker compared with these prior studies, which may be due to differences in study periods and prevalence of ART use.

Our assessment of ART conception as a potential mediator in the association between obesity and twin delivery is, to our knowledge, novel. Obesity is a known risk factor for subfertility and infertility,^31,32,42,43^ leading to increased use of IVF and other fertility treatments among women with overweight or obesity.^19^ In accordance with prior studies, we observed a modest increase in the frequency of ART conception ending in childbirth in women with overweight and obesity; however, this increased use of ART mediated only a small proportion of the association between obesity and twin delivery. Another possible reason for increased dizygotic twinning in women with high BMI is an increased prevalence of polycystic ovary syndrome, which is associated with use of fertility drugs^44^ that increase the risk of multifetal pregnancy.^45^

There are other possible factors that may contribute to the results found in our study. First, some women may undergo a selective reduction in twins, which is typically carried out around 10 weeks’ gestation.^46^ It is possible that selective reduction occurs at higher rates in women with obesity who conceived by ART who may have other comorbidities that would jeopardize the continuation of twin pregnancy to the live birth of both twins. This could explain the lower rates of twin deliveries observed in women with obesity class I vs those with normal BMI. Second, in some settings, women with a very high BMI are not eligible for publicly funded ART or are advised not to pursue ART due to a lower perceived success rate.^47,48,49^ Such restrictions have been disputed,^42^ and pooled data from several studies suggest that obesity alone is not sufficient to withhold treatment.^43^ ART practices in British Columbia do not follow explicit restrictions, but IVF access for women with a BMI of 40 or greater is restricted to hospital-based programs, as opposed to outpatient fertility clinics. It is, therefore, possible that some women with obesity are advised against pursuing ART until their BMI improves. This could explain our differential findings in women with class III obesity.

### Strengths and Limitations

The strengths of this study include large population-level data including all births in British Columbia, Canada, minimizing possible selection bias. The medical record ascertainment of ART conception was consistent throughout the study period.

The study also has several limitations. First, prepregnancy BMI had a sizeable proportion of missing data. Multiple imputation results showed little difference from the complete-case analyses, indicating that our results are valid under a missing at random assumption. We further investigated the validity of our findings under several non–missing at random scenarios. In each of these scenarios, the association between high BMI and twinning was maintained, but the degree of mediation by ART ranged from 0 to more than 50%. Second, we lacked information on chorionicity. The analysis of opposite-sex twins, which we used as a proxy to restrict to dichorionic twins, was congruent with the findings from previous studies of the association between obesity and dichorionic twinning.^18^ Third, we did not have data on pregnancies ending prior to 20 weeks’ gestation (miscarriages) and thus could not assess the true incidence of twin pregnancy. Twin pregnancy and obesity are both known risk factors for miscarriage in spontaneous and ART-conceived pregnancies,^50,51^ which could lead to a left-truncation bias. We performed quantitative bias analysis to address this bias, assuming moderate differences in the rates of miscarriage by BMI groups and found similar results to our primary analyses. However, if the degree of differential pregnancy loss was higher than we assumed, our findings may be biased. Fourth, several studies show that self-reported BMI tends to underreport overweight and obesity.^52,53,54^ Sensitivity analysis adjusting for such misclassification showed similar results to our primary analysis, suggesting that our results are robust in this respect. Fifth, the association between BMI and twin delivery in our study may be susceptible to unmeasured confounding, as evidenced by our relatively low estimated E-values. We did not have information about race and ethnicity, which is a possible confounder. Furthermore, the use of BMI as a measure of adiposity-related body composition in different populations has been questioned, and therefore, further research related to our findings should be conducted in diverse populations and stratified by race and ethnicity. We were also unable to link subsequent deliveries to the same women over the study period. Additionally, women with class III obesity may have a higher rate of fertility drug use, which may explain the elevated rate of twin birth in this group.

## Conclusions

In this cohort study of 524 845 deliveries, we found that women with overweight and obesity had higher rates of twin delivery; however, the proportion of twins attributed to elevated prepregnancy BMI was relatively low. Women with higher BMI used ART at higher rates than those with normal BMI, and this partially mediated the association between high prepregnancy BMI and twin delivery in those with overweight or class I or II obesity. However, we did not find any evidence of mediation by ART in women with class III obesity, who had the highest rates of twin delivery.

## Notes

Supplement 1. **eTable 1.** Comparison of Women With and Without Information on Prepregnancy BMI **eTable 2.** Rate of Twin Pregnancies of Same and Opposite Sex by BMI Group **eAppendix.** Statistical Details of Sensitivity Analyses **eTable 3. **Complete Case Analysis **eTable 4.** Results of Mediation Analysis Based on Imputation Assuming 4 Missing Data Scenarios **eTable 5.** Results of Sensitivity Analysis for Body-Mass-Index Misclassification Compared With Primary Results (Complete Cases) **eTable 6.** Additional Results of Sensitivity Analysis for Body Mass Index Misclassification Compared With Primary Results (Complete Cases) **eFigure 1.** Directed Acyclic Graph Representing Mediation Model **eFigure 2.** Trends in Twin Rates Among ART and Non-ART Conceptions During the Study Period **eFigure 3.** Inclusion of Women in Study **eFigure 4.** Rates of Twin Pregnancy by Prepregnancy BMI Stratified by ART Conception

Supplement 2.
Data Sharing Statement
