# Supplementary material for: Obesity, Twin Pregnancy, and the Role of Assisted Reproductive Technology
Source: JAMA Netw Open. 2024 Jan 9;7(1):e2350934. doi: 10.1001/jamanetworkopen.2023.50934 (PMC10777255; doi:10.1001/jamanetworkopen.2023.50934)
Supplement: Supplement 1. — eTable 1. Comparison of Women With and Without Information on Prepregnancy BMI eTable 2. Rate of Twin Pregnancies of Same and Opposite Sex by BMI Group eAppendix. Statistical Details of Sensitivity Analyses eTable 3. Complete Case Analysis eTable 4. Results of Mediation Analysis Based on Imputation Assuming 4 Missing Data Scenarios eTable 5. Results of Sensitivity Analysis for Body-Mass-Index Misclassification Compared With Primary Results (Complete Cases) eTable 6. Additional Results of Sensitivity Analysis for Body Mass Index Misclassification Compared With Primary Results (Complete Cases) eFigure 1. Directed Acyclic Graph Representing Mediation Model eFigure 2. Trends in Twin Rates Among ART and Non-ART Conceptions During the Study Period eFigure 3. Inclusion of Women in Study eFigure 4. Rates of Twin Pregnancy by Prepregnancy BMI Stratified by ART Conception [file jamanetwopen-e2350934-s001.pdf]

Supplemental Online Content

Bone JN, Joseph KS, Magee LA, et al. Obesity, twin pregnancy, and the role of assisted reproductive technology. *JAMA Netw Open.* 2024;7(1):e2350934.
doi:10.1001/jamanetworkopen.2023.50934

eTable 1. Comparison of Women With and Without Information on Prepregnancy BMI

eTable 2. Rate of Twin Pregnancies of Same and Opposite Sex by BMI Group

eAppendix. Statistical Details of Sensitivity Analyses

eTable 3. Complete Case Analysis

eTable 4. Results of Mediation Analysis Based on Imputation Assuming 4 Missing Data Scenarios

eTable 5. Results of Sensitivity Analysis for Body-Mass-Index Misclassification Compared With Primary Results (Complete Cases)

eTable 6. Additional Results of Sensitivity Analysis for Body Mass Index Misclassification Compared With Primary Results (Complete Cases)

eFigure 1. Directed Acyclic Graph Representing Mediation Model

eFigure 2. Trends in Twin Rates Among ART and Non-ART Conceptions During the Study Period

eFigure 3. Inclusion of Women in Study

eFigure 4. Rates of Twin Pregnancy by Prepregnancy BMI Stratified by ART Conception

This supplemental material has been provided by the authors to give readers additional information about their work.

eTable 1. Comparison of Women With and Without Information on Prepregnancy BMI

	**Complete BMI (N = 392,046)**	**Missing BMI (N = 132,799)**
**Maternal age**		
Median [IQR]	31.5 [27.9;34.9]	31.2 [27.2;35.0]
< 20	7195 (1.84)	3865 (2.91)
20-24	39867 (10.2)	16344 (12.3)
25-34	247311 (63.1)	78787 (59.3)
35-39	80053 (20.4)	26974 (20.3)
≥40	17620 (4.49)	6829 (5.14)
**Parity**		
Nulliparous	189557 (48.4)	53886 (40.6)
1	139007 (35.5)	50679 (38.2)
≥2	63444 (16.2)	28163 (21.2)
**Maternal height**		
Median [IQR]	165 [160;170]	164 [160;168]
< 165	194536 (49.6)	21650 (52.4)
165-168	93919 (24.0)	9548 (23.1)
169-172	46715 (11.9)	4658 (11.3)
≥173	56876 (14.5)	5495 (13.3)
**Smoking**		
Yes	26424 (6.74)	10470 (7.88)
No	365622 (93.3)	122329 (92.1)
**Use of ART**		
Yes	11523 (2.94)	3872 (2.92)
No	380523 (97.1)	128927 (97.1)
**Twin pregnancy**		
Yes	5813 (1.48)	2482 (1.87)
No	386233 (98.5)	130317 (98.1)

IQR = interquartile range
ART = assisted reproductive technology

© 2024 Bone JN et al. *JAMA Network Open.*

eTable 2. Rate of Twin Pregnancies of Same and Opposite Sex by BMI Group

**Pre-pregnancy BMI**	Total deliveries	N twin births overall (per 1000)	N twin births same sex (per 1000)	N twin births opposite sex (per 1000)
Underweight	22396	253 (11.3)	182 (8.13)	71 (3.2)
Normal	231583	3323 (14.4)	2221 (9.59)	1102 (4.8)
Overweight	83887	1340 (16.0)	873 (10.41)	467 (5.6)
Obese I	33263	531 (16.0)	334 (10.1)	197 (5.9)
Obese II	13308	222 (16.7)	136 (10.2)	86 (6.5)
Obese III	7609	144 (18.9)	92 (12.1)	52 (6.8)

© 2024 Bone JN et al. *JAMA Network Open.*

eAppendix. Statistical Details of Sensitivity Analyses
The main text refers to five primary sensitivity analyses, details of which are as follows:

First, we repeated the analyses in the subset of women with twins of opposite sex, as this subset would include only dizygotic twins, which have a stronger association with environmental and maternal factors (such as BMI). Although we did not have information on zygocity and chorionicity for twin deliveries, opposite-sex twins constitute approximately half of all dizygotic twins (per Weinberg's rule (1)). This analysis assumed that the same-sex and opposite-sex twin delivery ratio was not affected by pre-pregnancy BMI and all other factors, and that the analysis of opposite-sex twin deliveries would approximate the results for all dizygotic twin births. These results are summarized in Table 4 of the main text.

Second, we performed complete-case analyses of records with known BMI to assess possible selection bias (Table S3 below).

Third, we further carried out deterministic analyses for the missing data under four other missing-data assumptions. The first two scenarios corresponded to 'worst case' selection biases for the proportion mediated and assumed that all ART pregnancies with missing BMI were from women with obesity while all other women with missing data had normal BMI (scenario 1) and vice versa (scenario 2). The other two scenarios corresponded to 'worst case' selection biases for the total effect of BMI on twinning and assumed that all missing BMI values (regardless of ART status) were from women with obesity (scenario 3) or were from women with normal BMI (scenario 4). Specifically:

a) Scenario 1 –we assumed that all ART pregnancies missing BMI were obese with relative proportions of class I, II and III the same as in observed cases, while all others missing BMI had normal BMI.
b) Scenario 2- we assumed that all ART pregnancies missing BMI were normal weight, while all others missing BMI had the same were obese with relative proportions of class I, II and III the same as in observed cases.
c) Scenario 3 – in this scenario we assumed that all missing BMI values, regardless of ART status were obese with relative proportions of class I, II and III the same as in observed cases.
d) Scenario 4 – in this scenario we assumed that the pregnancies missing BMI were those with normal BMI.

These results are summarized in Table S4 below.

Third, we repeated the mediation analyses adjusting for various levels of possible misclassification of BMI (2) as self-reported BMI is known to underestimate true BMI (3–5). There have been numerous studies indicating that self-reported BMI differs from clinical estimates, with most studies finding that self-reporting tends to underestimate BMI (3–5). This under-reporting may lead to misclassification of cases, e.g., some women listed as 'normal' actually being overweight, and some who are obese being listed as 'normal'. We conducted this sensitivity analysis to assess this possible issue, assuming that most misclassification would be downwards (i.e., obese to overweight or overweight to normal) and that very few underweight women were misclassified as a higher BMI (2).

We used the following misclassification matrix where cells represent probability of misclassification (rounded). Due to high computation time and similarity between multiply imputed and complete case

© 2024 Bone JN et al. *JAMA Network Open.*

results we conducted these analyses only on complete cases. Confidence intervals were calculated from 100 bootstrap resamples. Results are summarized in Table S5.

	Underweight	Normal	Overweight	Obese I	Obese II	Obese III
Underweight	0.95	0.05	< 0.001	< 0.001	< 0.001	< 0.001
Normal	0.01	0.94	0.05	< 0.001	< 0.001	< 0.001
Overweight	< 0.001	0.01	0.94	0.05	< 0.001	< 0.001
Obese I	< 0.001	< 0.001	0.01	0.94	0.05	< 0.001
Obese II	< 0.001	< 0.001	< 0.001	0.01	0.94	0.05
Obese III	< 0.001	< 0.001	< 0.001	< 0.001	0.01	0.99

Fifth, our cohort only included pregnancies lasting to ≥20 weeks gestation. As has been documented in previous methodological studies, this can create a possible left-truncation bias if there is differential early pregnancy loss between groups. In this case, if obese women were more likely to miscarry before 20 weeks than those of normal BMI, then the association between BMI and twin pregnancy observed in our cohort would be biased. To assess the sensitivity of our primary result to this we conducted a probabilistic quantitative bias analysis assuming that the selection bias odds between higher levels of BMI and normal BMI women decreased with increasing. We assumed triangular distribution with moderate amount of left truncation bias (see table below); 1.0 represents no left-truncation bias while smaller values represent higher degrees of bias. The bias analysis was run 100,000 times and we report the median bias adjusted risk ratio with corresponding confidence interval taken as the 2.5th and 97.5th percentiles of the resulting distribution. Results are summarized in Table S6 below.

1. Fellman J, Eriksson AW. Weinberg's differential rule reconsidered. Hum Biol. 2006 Jun;78(3):253–75.

2. Küchenhoff H, Mwalili SM, Lesaffre E. A general method for dealing with misclassification in regression: the misclassification SIMEX. Biometrics. 2006 Mar;62(1):85–96.

3. Headen I, Cohen AK, Mujahid M, Abrams B. The accuracy of self-reported pregnancy-related weight: a systematic review. Obes Rev Off J Int Assoc Study Obes. 2017 Mar;18(3):350–69.

4. Han E, Abrams B, Sridhar S, Xu F, Hedderson M. Validity of Self-Reported Pre-Pregnancy Weight and Body Mass Index Classification in an Integrated Health Care Delivery System. Paediatr Perinat Epidemiol. 2016;30(4):314–9.

5. Bodnar LM, Abrams B, Bertolet M, Gernand AD, Parisi SM, Himes KP, et al. Validity of Birth Certificate-Derived Maternal Weight Data. Paediatr Perinat Epidemiol. 2014;28(3):203–12.

6. Lash TL, Fox MP, MacLehose RF, Maldonado G, McCandless LC, Greenland S. Good practices for quantitative bias analysis. Int J Epidemiol. 2014 Dec;43(6):1969–85.

© 2024 Bone JN et al. *JAMA Network Open.*

eTable 3. Complete Case Analysis

Pre-pregnancy BMI	Total effect aRR (95% CI)	Natural direct effect aRR (95% CI)	Natural indirect effect aRR (95% CI)	Proportion mediated (95% CI)
Underweight	0.84 (0.74, 0.96)	0.85 (0.75, 0.97)	0.99 (0.97, 1.00)	7 (-2, 15)
Normal	1.00 (ref)	1.00 (ref)	1.00 (ref)	-
Overweight	1.14 (1.07, 1.22)	1.13 (1.05, 1.20)	1.01 (1.01, 1.02)	10 (3, 17)
Obese I	1.17 (1.07, 1.29)	1.14 (1.03, 1.25)	1.03 (1.02, 1.04)	20 (7, 33)
Obese II	1.20 (1.05, 1.39)	1.16 (1.00, 1.33)	1.04 (1.02, 1.06)	23 (3, 43)
Obese III	1.45 (1.22, 1.71)	1.48 (1.25, 1.75)	0.98 (0.96, 1.00)	-7 (-14, 0)

aRR = adjusted risk ratio.

Effects are estimated from mediation analyses pooled across 20 multiply imputed data sets.

Results are adjusted for maternal height, age, smoking status, parity and fiscal year.

E-values represent strength of unmeasured confounder (on rate ratio scale) needed to bring the point estimate for total effects to 1.0.

© 2024 Bone JN et al. *JAMA Network Open.*

eTable 4. Results of Mediation Analysis Based on Imputation Assuming 4 Missing Data Scenarios

	**Total effect aRR (95% CI)**					**Natural direct effect aRR (95% CI)**					**Natural indirect effect for (95% CI)**					**Proportion mediated (95% CI)**				
	Primary analysis	Scenario 1	Scenario 2	Scenario 3	Scenario 4	Primary analysis	Scenario 1	Scenario 2	Scenario 3	Scenario 4	Primary analysis	Scenario 1	Scenario 2	Scenario 3	Scenario 4	Primary analysis	Scenario 1	Scenario 2	Scenario 3	Scenario 4
Underweight	0.84 (0.74, 0.95)	0.80 (0.71, 0.92)	0.81 (0.71, 0.93)	0.84 (0.74, 0.96)	0.78 (0.69, 0.89)	0.85 (0.75, 0.96)	0.80 (0.70, 0.91)	0.84 (0.74, 0.96)	0.85 (0.75, 0.97)	1.07 (0.97, 1.17)	0.99 (0.98, 1.00)	1.01 (1.00, 1.02)	0.96 (0.95, 0.98)	0.99 (0.97, 1)	1.03 (1.02, 1.04)	6 (-2, 13)	-4 (-10, 3)	17 (4, 30)	6 (2,15)	6 (0, 11)
Normal	1.00 (ref)	1.00 (ref)	1.00 (ref)	1.00 (ref)	1.00 (ref)	1.00 (ref)	1.00 (ref)	1.00 (ref)	1.00 (ref)	1.00 (ref)	1.00 (ref)	1.00 (ref)	1.00 (ref)	1.00 (ref)	1.00 (ref)	-	-	-	-	-
Overweight	1.14 (1.07, 1.21)	1.09 (1.03, 1.17)	1.10 (1.03, 1.18)	1.14 (1.07, 1.22)	1.06 (1.00, 1.13)	1.12 (1.05, 1.19)	1.06 (1.00, 1.14)	1.12 (1.04, 1.19)	1.13 (1.06, 1.21)	1.05 (0.99, 1.12)	1.01 (1.01, 1.02)	1.03 (1.02, 1.04)	0.99 (0.98, 1.00)	1.01 (1.01, 1.02)	1.01 (1.00, 1.02)	11 (3, 18)	32 (9, 56)	-11 (-21, -1)	9 (3, 16)	15 (-3,33)
Obese I	1.16 (1.06, 1.27)	1.56 (1.34, 1.83)	1.30 (1.13, 1.49)	1.47 (1.30, 1.68)	1.09 (1.00, 1.20)	1.12 (1.03, 1.23)	1.42 (1.20, 1.67)	1.41 (1.23, 1.61)	1.48 (1.3, 1.68)	1.07 (0.97, 1.17)	1.03 (1.02, 1.04)	1.10 (1.06, 1.14)	0.92 (0.89, 0.96)	1 (0.98, 1.01)	1.03 (1.02, 1.04)	23 (7, 39)	26 (13, 38)	-36 (-61, -11)	-1 (-5, 3)	30 (-2, 63)
Obese II	1.17 (1.02, 1.34)	1.33 (1.17, 1.51)	1.23 (1.11, 1.38)	1.40 (1.26, 1.55)	1.12 (0.98, 1.29)	1.13 (0.98, 1.29)	1.11 (0.96, 1.27)	1.30 (1.17, 1.44)	1.36 (1.23, 1.51)	1.08 (0.94, 1.25)	1.04 (1.02, 1.06)	1.20 (1.15, 1.25)	0.95 (0.94, 0.97)	1.03 (1.01, 1.04)	1.04 (1.02, 1.06)	27 (0, 54)	68 (36, 100)	-27 (-43, -10)	9 (4, 13)	32 (-8, 73)
Obese III	1.41 (1.19, 1.66)	1.29 (1.18, 1.40)	1.17 (1.08, 1.26)	1.30 (1.20, 1.39)	1.35 (1.14, 1.60)	1.44 (1.22, 1.69)	1.10 (1.00, 1.21)	1.22 (1.14, 1.32)	1.26 (1.17, 1.35)	1.38 (1.17, 1.64)	0.98 (0.96, 1.00)	1.17 (1.14, 1.20)	0.95 (0.94, 0.96)	1.03 (1.02, 1.04)	0.98 (0.96, 0.99)	-7 (-15, 0)	64 (41, 87)	-33 (-51, -15)	12 (8, 17)	-9 (-17,0)

aRR = adjusted risk ratio

© 2024 Bone JN et al. *JAMA Network Open.*

eTable 5. Results of Sensitivity Analysis for Body-Mass-Index Misclassification Compared With Primary Results (Complete Cases)

	**Total effect aRR (95% CI)**		**Natural direct effect aRR (95% CI)**		**Natural indirect effect for (95% CI)**		**Proportion mediated % (95% CI)**	
	Primary analysis	Measurement error adjusted	Primary analysis	Measurement error adjusted	Primary analysis	Measurement error adjusted	Primary analysis	Measurement error adjusted
Underweight	0.84 (0.74, 0.95)	0.81 (0.67, 0.96)	0.85 (0.75, 0.96)	0.81 (0.68, 0.97)	0.99 (0.98, 1.00)	0.99 (0.99, 1.00)	6 (-2, 13)	2 (-10, 6)
Normal	1.00 (ref)	1.00 (ref)	1.00 (ref)	1.00 (ref)	1.00 (ref)	1.00 (ref)	-	1.00 (ref)
Overweight	1.14 (1.07, 1.21)	1.17 (1.08, 1.27)	1.12 (1.05, 1.19)	1.16 (1.07, 1.26)	1.01 (1.01, 1.02)	1.01 (1.00, 1.01)	11 (3, 18)	3 (1, 8)
Obese I	1.16 (1.06, 1.27)	1.18 (1.05, 1.32)	1.12 (1.03, 1.23)	1.16 (1.03, 1.30)	1.03 (1.02, 1.04)	1.01 (1.00, 1.02)	23 (7, 39)	8 (1, 15)
Obese II	1.17 (1.02, 1.34)	1.20 (1.01, 1.43)	1.13 (0.98, 1.29)	1.18 (0.99, 1.40)	1.04 (1.02, 1.06)	1.02 (1.01, 1.03)	27 (0, 54)	9 (-1, 20)
Obese III	1.41 (1.19, 1.66)	1.54 (1.27, 1.87)	1.44 (1.22, 1.69)	1.56 (1.29, 1.89)	0.98 (0.96, 1.00)	0.99 (0.98, 1.00)	-7 (-15, 0)	-3 (-5, 0)

© 2024 Bone JN et al. *JAMA Network Open.*

eTable 6. Additional Results of Sensitivity Analysis for Body Mass Index Misclassification Compared With Primary Results (Complete Cases)

		Total effect aRR (95% CI)	
	Mode of bias odds (lower, upper limits) for triangular distribution	Primary analysis	Left-truncation bias adjusted
Underweight	1.00 (0.90. 1.10)	0.84 (0.74, 0.95)	0.79 (0.68, 0.91)
Normal	-	1.00 (ref)	1.00 (ref)
Overweight	0.98 (0.86, 1.00)	1.14 (1.07, 1.21)	1.18 (1.08, 1.30)
Obese I	0.96 (0.84, 1.00)	1.16 (1.06, 1.27)	1.19 (1.07, 1.35)
Obese II	0.94 (0.82, 1.00)	1.17 (1.02, 1.34)	1.27 (1.08, 1.49)
Obese III	0.92 (0.80, 1.00)	1.41 (1.19, 1.66)	1.46 (1.21, 1.77)

© 2024 Bone JN et al. *JAMA Network Open.*

eFigure 1. Directed Acyclic Graph Representing Mediation Model

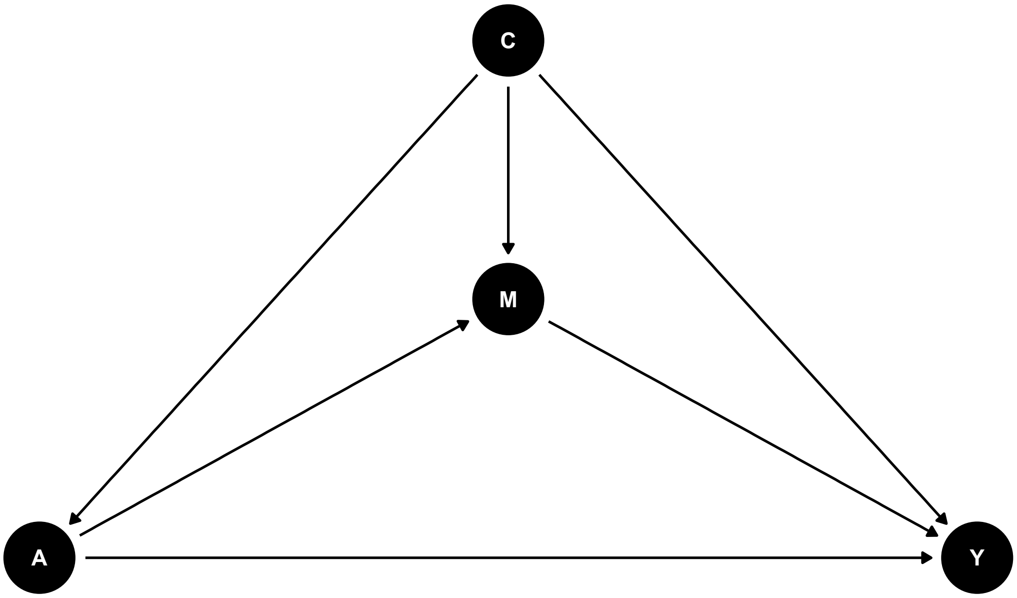

A (exposure): BMI category
M (mediator): ART
Y (outcome): Twin pregnancy
C (confounders not affected by the exposure): Age, Parity, Smoking, Height, Fiscal year

© 2024 Bone JN et al. *JAMA Network Open.*

eFigure 2. Trends in Twin Rates Among ART and Non-ART Conceptions During the Study Period

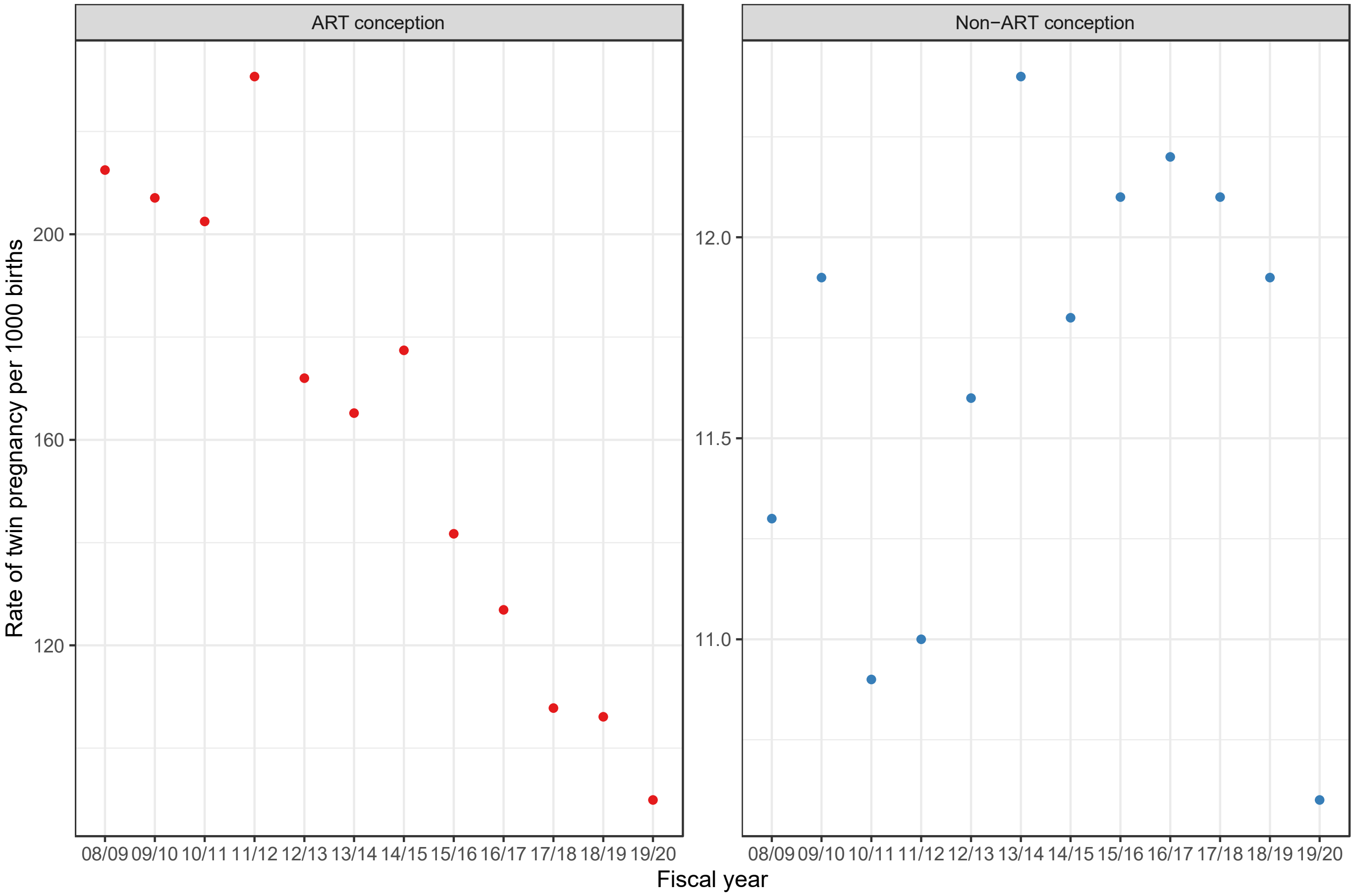

© 2024 Bone JN et al. *JAMA Network Open.*

eFigure 3. Inclusion of Women in Study

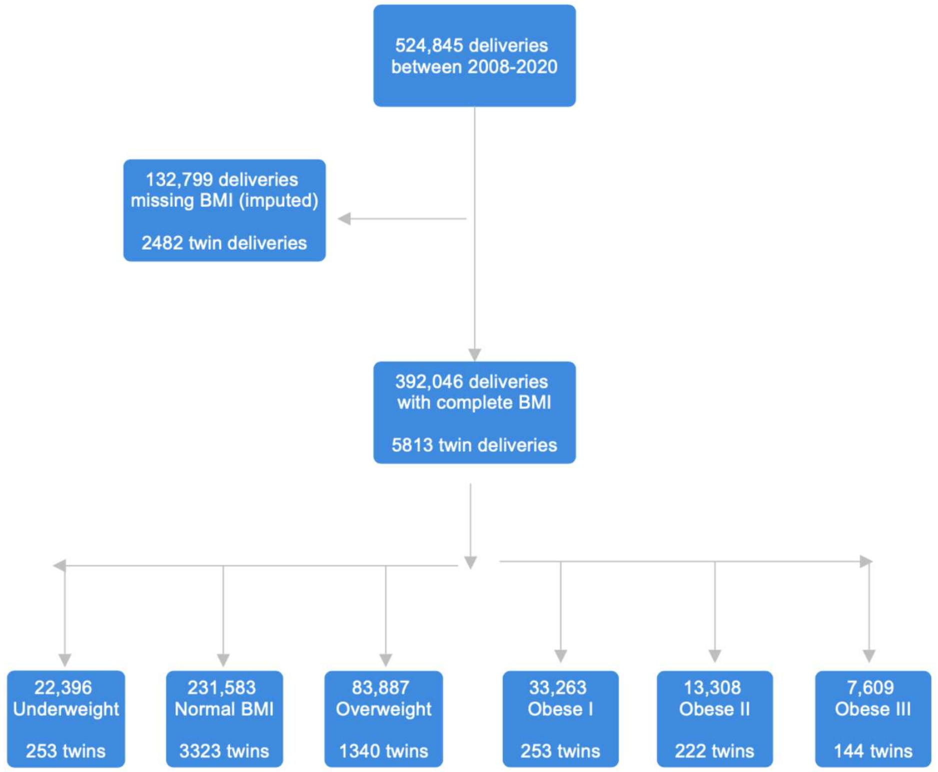

© 2024 Bone JN et al. *JAMA Network Open.*

eFigure 4. Rates of Twin Pregnancy by Prepregnancy BMI Stratified by ART Conception

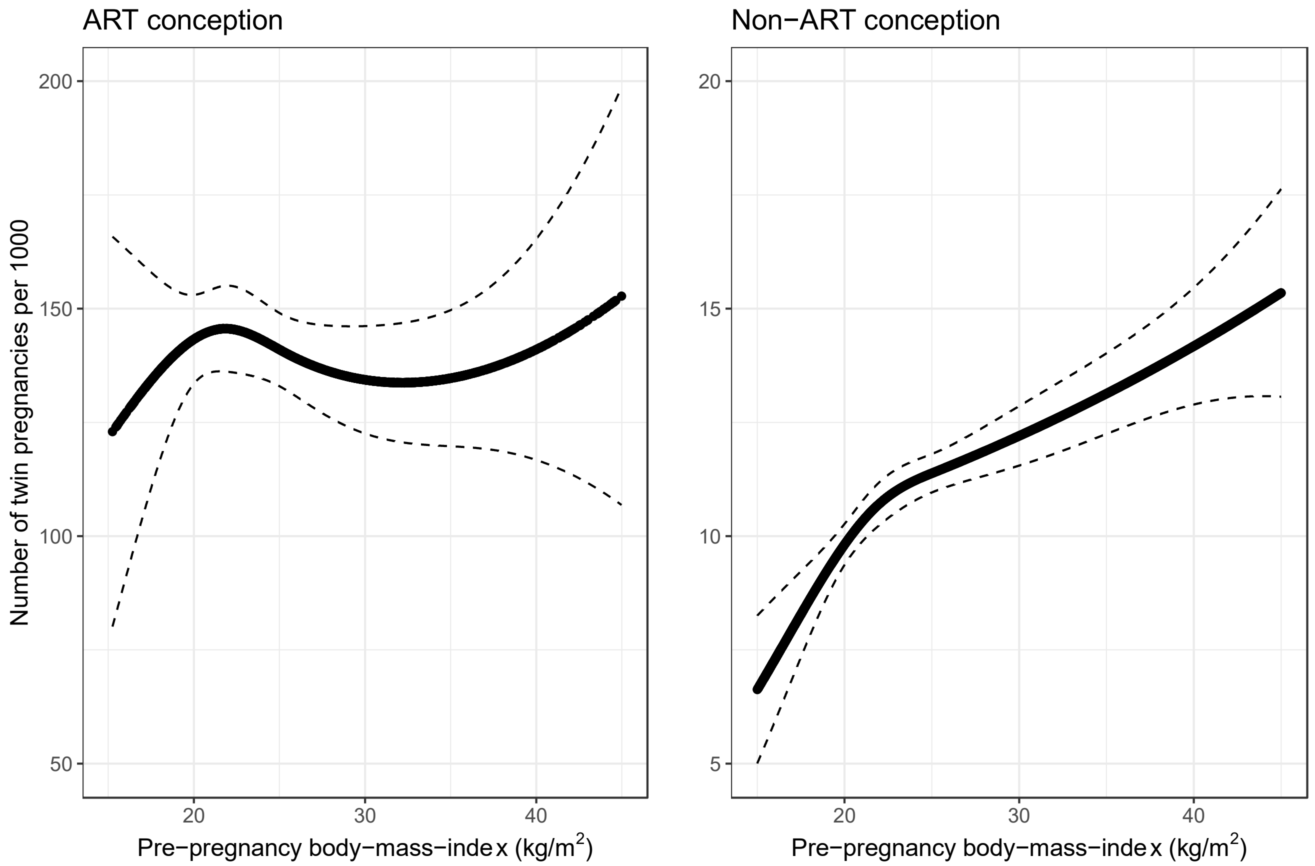

© 2024 Bone JN et al. *JAMA Network Open.*
