# Supplementary material for: Obesity, Twin Pregnancy, and the Role of Assisted Reproductive Technology
Source: JAMA Netw Open. 2024 Jan 9;7(1):e2350934. doi: 10.1001/jamanetworkopen.2023.50934 (PMC10777255; doi:10.1001/jamanetworkopen.2023.50934)
Supplement: Supplement 2. — Data Sharing Statement [file jamanetwopen-e2350934-s002.pdf]

Data Sharing Statement

Bone. Obesity, Twin Pregnancy, and the Role of Assisted Reproductive Technology. *JAMA Netw Open*. Published January 09, 2024. doi:10.1001/jamanetworkopen.2023.50934

Data
Data available: No

Additional Information
Explanation for why data not available: This data is property of Population Data British Columbia and is available from them upon request.
